# Phase Heterogeneity in Cholesterol-Containing Ternary Phospholipid Lamellar Phases

**DOI:** 10.1021/acsomega.2c04914

**Published:** 2023-02-08

**Authors:** Deborah L. Gater, Keontré I. Hughes, Vivian Stojanoff, Abdel F. Isakovic

**Affiliations:** † University College London, London WC1E 6BT, U.K.; ‡ Colgate University, Hamilton, New York 13346-1338, United States; § Michigan State University, East Lansing, Michigan 48824-1312, United States; ∥ Brookhaven National Laboratory, Upton, New York 11973-5000, United States

## Abstract

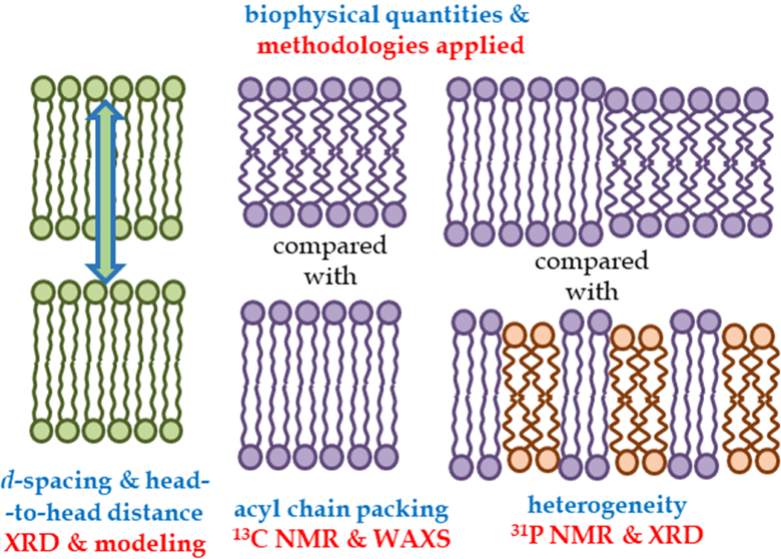

Pseudo-ternary mixtures of lamellar phase phospholipids (DPPC and brain sphingomyelin with cholesterol) were studied below *T*_m_ while comparing the influence of cholesterol content, temperature, and the presence of small quantities of vitamin D binding protein (DBP) or vitamin D receptor (VDR). The measurements, conducted by X-ray diffraction (XRD) and nuclear magnetic resonance (NMR), cover a range of cholesterol concentrations (20% mol. wt to 40% mol. wt.) and physiologically relevant temperature range (294–314 K). In addition to rich intraphase behavior, data and modeling are used to approximate the lipids’ headgroup location variations under the abovementioned experimental conditions.

## Introduction

Lamellar phase coexistence in ternary phospholipid systems containing cholesterol is a well-established phenomenon.^1^ In particular, the putative involvement of cholesterol and sphingomyelin in “lipid rafts,” or physiologically relevant domain formation/lateral lipid organization *in vivo*, has resulted in a wealth of study in this area (see the following for a more comprehensive background^2,3^). However, much of this work has been conducted on fluid bilayers, and less is understood about model lipid systems below their gel–fluid melting transition temperature (*T*_m_). The responses of ternary systems containing 1,2-dipalmitoyl-*sn*-glycero-3-phosphocholine (DPPC), sphingomyelin (SM), and cholesterol (Chol) systems to neuroleptic drugs^4^ and to phenothiazine derivatives^5^ have been studied at lower Chol and SM concentrations than studied here. Evidence for microdomain formation, based on asymmetry in the phase transition observed by differential scanning calorimetry (DSC), was reported in large unilamellar vesicles (LUVs) containing DPPC/16:0-SM/Chol (85:10:5 mol %).^4^ Similar evidence for phase separation was less clear in systems containing either 5 or 10% of both egg-yolk SM and Chol in DPPC, although the focus of that work was predominantly on inducing phase separation around the main DPPC transition temperature by the addition of phenothiazine derivatives.^5^ In both studies, the addition of small quantities (≤20 mol % total) of cholesterol and SM suppressed the DPPC pretransition in DSC and decreased the DPPC *T*_m_ (37.8 °C^6^) by ∼1 °C.

Here, we present an analysis of X-ray diffraction (XRD) data obtained for multilamellar vesicles (MLVs) comprising different proportions of DPPC, brain sphingomyelin (bSM), and Chol at three different temperatures between room temperature and physiological temperature (i.e., below or approaching the anticipated *T*_m_), with different combinations of buffer and added protein. The data are by nature complex for such systems, and thus we have sought to apply the methods of Harper et al.^7^ and of Rappolt^8^ to estimate the range of structural parameters exhibited in these conditions. The XRD analysis is supported by ^31^P and ^13^C solid-state nuclear magnetic resonance (NMR) spectroscopy data.

The choice of added protein–vitamin D receptor (VDR) or vitamin D binding protein (DBP) was motivated by a desire to commence a preliminary investigation of how lipid composition might affect the interactions of such proteins with phospholipid lamellar phases and thus influence the transport and availability of vitamin D within the body.^9^ There have been numerous studies investigating the *in vitro* and *in vivo* relationships between, for example, vitamin D metabolism and phospholipid organization,^10^ or vitamin D bioavailability, and factors including food/supplement matrix.^11^ However, a detailed review of this area is beyond the scope of this article. The present work may be seen to complement previous studies that have investigated the properties of VitD-containing lipid systems^12,13^ and studies reviewed in.^14^

DBP is a soluble globular protein related to the albumin family. DBP acts as a transport protein, binding up to 85% of circulating VD and its various metabolites.^15,16^ The interactions of VD, DBP (and metabolites of both) with membranes of different tissues have not yet been fully elucidated, although it has been suggested that it is “free” VitD that interacts with many cell membranes, rather than DBP-bound VitD, except in the case of specific tissues for which transport systems have been identified.^16,17^

In contrast, VDR is a nuclear receptor involved primarily in calcium and phosphate homeostasis and is expressed widely in a variety of tissue types.^18^ The activities of VDR are primarily genomic,^19^ although in some studies, VDR has also been colocated with the plasma membrane and with caveolin.^20^

## Materials and Methods

### Materials

(DPPC), d_62_-DPPC and porcine bSM were bought from Avanti Polar Lipids (Alabama). Chol and D_2_O were purchased from Sigma-Aldrich. Deionized water was used as a solvent for all sample preparation unless otherwise specified. Vitamin D binding protein (DBP, Globulin GC) was purchased from Athens Research (Georgia), and vitamin D receptor (VDR) was from Abcam (U.K.). All materials were used without further purification.

### Sample Preparation

MLVs were formulated at desired mole percent ratios of DPPC, d_62_-DPPC, SM, and Chol. MLVs were prepared by the thin-film hydration method, where the blend of lipids was weighed out accurately and first dissolved together in a mixture of chloroform and methanol. The solvent was removed using a dry nitrogen stream, and the samples were kept in vacuum overnight. The resulting thin film was then hydrated in deionized water. The hydrated samples were flash-frozen using liquid nitrogen and lyophilized overnight. Dry samples were stored at −20 °C until needed. Samples were hydrated by the addition of H_2_O as indicated for characterization by XRD or NMR. The solvent in excess (66 wt %) was added directly to the lipids above 70 °C. After this, samples were heat-cycled twice between 70 °C and frozen, allowed to warm slowly to room temperature and equilibrate overnight, and were briefly vortexed before analysis. Briefly, the choice of 66 wt % water was made to balance the need for sufficient sample concentration for analysis with a desire to ensure there was sufficient excess water to avoid any hydration effect on d-spacing (i.e., the ratio of hydration water, *R*_W,_ was >50^21,22^). Vitamin D binding protein (DBP) was purchased from Athens Research, and vitamin D receptor (VDR) was purchased from Abcam. Proteins were reconstituted according to the manufacturer’s instructions, and aliquots of the dissolved protein were added to the hydrated lipid samples to give lipid/protein ratios of the order of 30,000:1.

### NMR Spectra Acquisition, Processing, and Analysis

All NMR data were acquired on a 200 MHz, wide bore Bruker (Karlsruhe, Germany) spectrometer operating at 4.7 T with a ^1^H resonance of 200.1 MHz, a ^13^C resonance of 50.3 MHz, and a ^31^P resonance of 81.0 MHz. ^13^C NMR magic angle spinning (MAS) spectra were recorded in a 4 mm two-channel cross polarization-MAS probe with a spinning frequency of 5.5 kHz using a standard single-pulse program with a ^13^C 90° pulse of 2.0 μs at 80 W. Power-gated ^1^H decoupling was applied at 10 W. A recycle delay of 2.0 s was used, 8192 scans were acquired for all spectra, and line broadening of 5 Hz was applied. ^13^C spectra chemical shifts were internally calibrated to the acyl methyl carbon resonance (note that an alternative internal calibration to the choline C_γ_ instead did not alter any of the observations reported here). Single-pulse ^31^P spectra were recorded in the same probe (under static conditions), with a 90° pulse of 2.0 μs at 24.6 W. The recycle delay was again 2.0 s, 2048 scans were acquired, and line broadening of either 50 or 75 Hz was applied. All data were analyzed using Bruker Topspin software v4.1.1 (Karlsruhe, Germany). ^31^P chemical shift anisotropy (CSA) parameters were extracted from fits performed with single nuclei (i.e., no overlapping CSAs) in the SOLA NMR plugin within Topspin.

### X-ray Acquisition

All data were acquired at the X6A beamline at NSLS at the Brookhaven National Laboratory (Long Island, NY, US). Data were recorded with a 350 mm detector distance, a slit size of 130×130 mm, and an energy of 9 keV. For each sample, three images were acquired in bin mode, with the oscillation range of 0.05°. Data were extracted from 2D using Fit2D (freely available software from ESRF) and converted to *d*-spacing using the standard expression.

### X-ray Electron Density Profiles

Electron density plots were obtained using the approach of Harper et al.^4^ and refined according to the method proposed by Rappolt^5^ and following Pabst et al.^23^ Appropriate lipid structural parameters were taken from Greenwood et al.^24^ with reference to Nagle et al.^25^

## Results and Discussion

**Figure 1:**
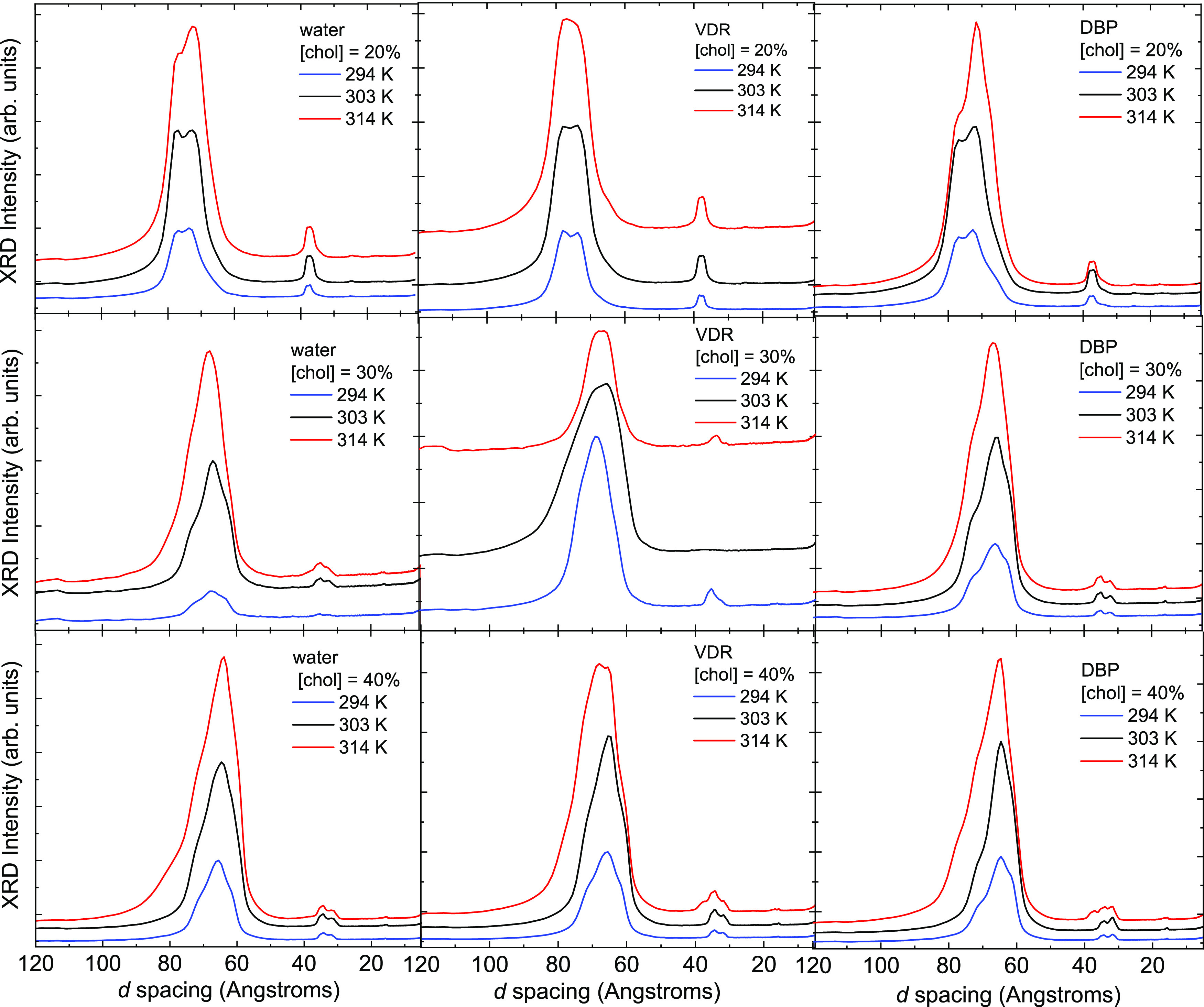
X-ray diffraction scans for all samples, cholesterol concentrations, and temperatures in this study. These are raw data and are plotted in “waterfall” mode for clarity.

Figure 1 displays raw X-ray diffraction (XRD) data for a variety of samples, temperature values, and cholesterol concentration values. Two significant features of all of the plotted XRD data are that each show (a) broadly lamellar structure with d-spacing in the range ∼60–80 Å and repeat spacings at 1/2 and 1/4 and (b) inhomogeneity in the sense that the first order peaks are both broad and often appear qualitatively to consist of two or more overlapping peaks. We are not aware of an exactly comparable system, but the d-spacing range observed here is consistent with previous reports for related lipid mixtures (e.g., close to C16:0-SM/Chol mixtures,^26^ and somewhat higher than 1,2-dioleoyl-*sn*-glycero-3-phosphocholine (DOPC)/DPPC/Chol mixtures or DPPC/Chol mixtures).^27^ Equally, the presence of substantial inhomogeneity is not unexpected. Although DPPC and SM have been reported to be miscible, the addition of Chol, particularly in ternary systems, is known to induce phase separation,^26,27^ and previous DSC studies of DPPC/SM/Chol systems (albeit with lower concentrations of SM and Chol) have also reported evidence of microdomain formation.^4^ In other ternary systems, phase coexistence below *T*_m_ in the range of Chol concentrations studied here is usually identified as a lamellar gel (*L*_β_) plus liquid-ordered (*L*_o_) coexistence.^1^

However, phase separation in ternary combinations of DPPC or SM with 5-cholesten-3-one and ceramide or Chol and 1,2-dipalmitoyl-*sn*-glycerol was identified as coexistence between an *L*_β_ and an intermediate phase with properties between those of the *L*_β_ and the *L*_o_ phases.^28^ Interestingly, in the same work, no evidence of phase coexistence was observed in systems containing either DPPC or SM with equimolar quantities of Chol and ceramide below *T*_m_. In similar but not quite analogous ternary systems containing either DPPC or SM with equimolar quantities of ceramide and cholesterol, evidence for gel phase coexistence was interpreted with respect to the balance of favorable H-bonding interactions between ceramide and Chol vs ceramide or Chol with SM.^29^ In this comparison between DPPC and SM, it was found that SM likely had a stronger interaction than DPPC with Chol/Cer. In our system, one could propose a similar heterogeneity arising from different H-bonding interactions between Chol and the SM or DPPC interfacial regions—particularly as a result of the SM −OH and −NH groups in the myelin backbone. Previous work provides further insight into interfacial H-bonding interactions of Chol^30^ and the complexities of Chol-SM H-bonding interactions.^31−33^

The heterogeneous nature of the samples we look at, coupled with the practical difficulty of taking a large number of XRD scans with a sufficiently small spot size for X-ray photons, contributes to the less-than-ideal XRD line shapes. Further study of these systems with micro- and nanoscale resolution, for example, using near-field optical and IR techniques^34^ or X-ray spectromicroscopy,^35^ may help to resolve outstanding questions relating to this heterogeneity.

**Figure 2:**
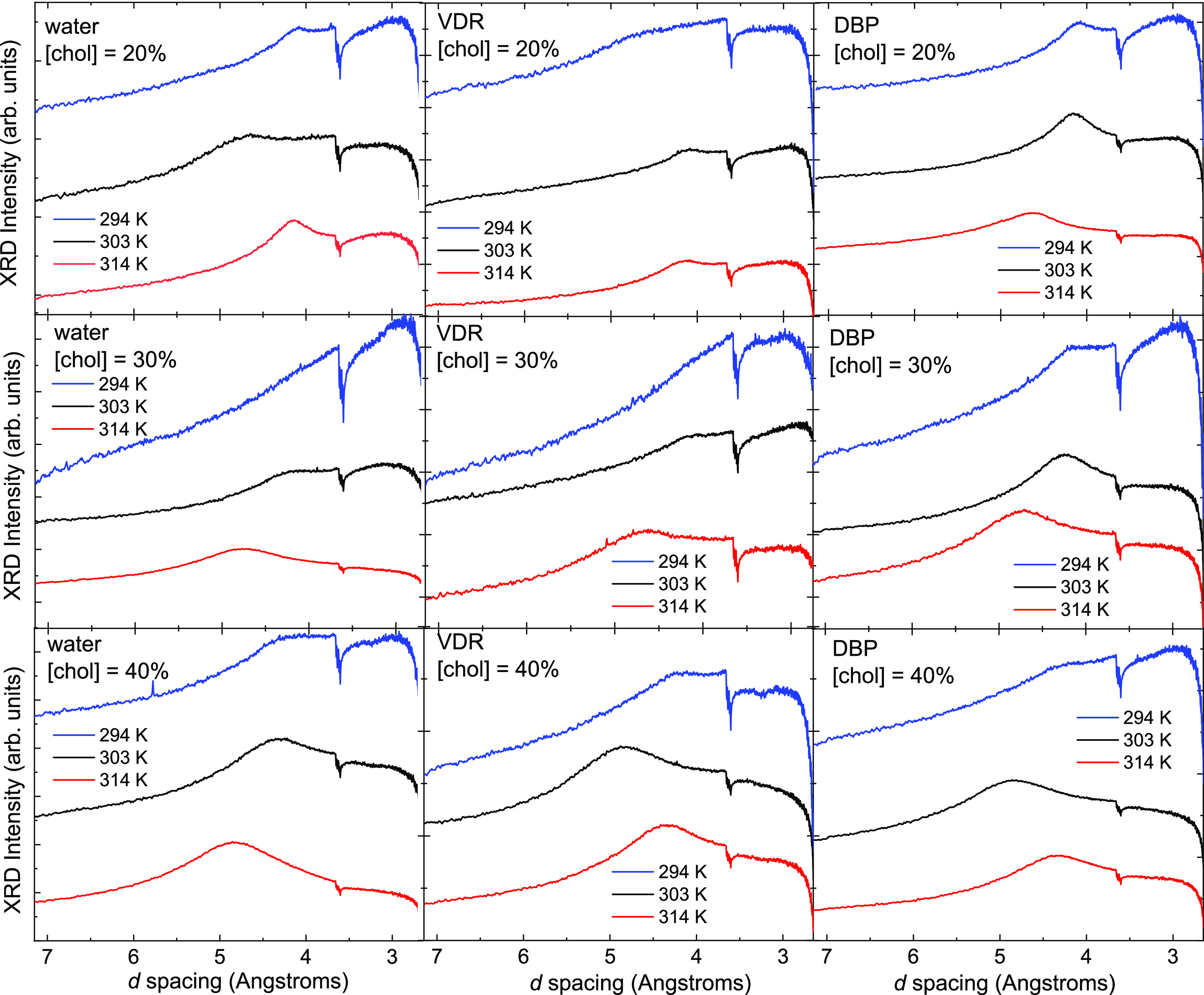
X-ray diffraction scans focusing on the wide-angle region (2.65–7.15 Å) for all samples, cholesterol concentrations, and temperatures in this study. These are raw extracts and are plotted in waterfall mode for clarity.

In Figure 2, we show XRD data for the purpose of examining evidence for/against the presence of crystalline cholesterol and to assess lateral chain packing in the WAXS region. Crystalline Chol (usually in its monohydrate form) has characteristic reflections at ∼34 Å^36^ as well as relatively sharp reflections in the wide-angle region (2–4 Å), which can be observed in coexistence with lamellar phase peaks if present in the sample (see Figure 2).^37^ In the data presented here, there is no evidence of crystalline Chol in the 20 mol % Chol samples, and there is an overlap between the second-order lamellar peaks at 30 and 40 mol % Chol and the region where Chol crystal reflections would appear.

At the same time, there is no evidence of crystalline Chol in the wide-angle region in any sample, although the region below ∼3.0 Å is toward the limit of instrumental detection in our setup. For most samples, the wide-angle region of the diffraction pattern shows a single broad, asymmetric peak between 4 and 5 Å that is characteristic of lateral acyl chain packing. Where the wide-angle peak can be resolved for the 20 and 30 mol % Chol samples at the two lower temperatures, this peak is centered between 4.1 and 4.2 Å, shifting to 4.6–4.9 Å at 314 K.

A similar dependence of the peak position corresponding to acyl chain lateral spacing on both cholesterol and temperature has been observed in DPPC/Chol systems, although significantly higher temperatures and cholesterol content were required to achieve a lateral spacing of 4.6 Å^38^ and the increased spacing may be a result of the unsaturated acyl chain components of bSM. It is notable that there is no overlap of peaks in the wide-angle region and that all peaks are broad. These observations suggest that in all conditions studied, any coexisting lamellar phases present exhibit substantial but similar acyl chain lateral packing—likely induced in part by Chol and in part by the unsaturated components and varying chain lengths of the bSM—and that the disorder of the chains increases over the 16 K temperature range from values associated with the *L*_β_ phase to values that are associated with the extent of disorder present in the *L*_o_ or even *L*_α_ phases,^39^ although there is no substantial change in *d*-spacing over the same temperature range. A recent study of chain melting in SM bilayers reported that different segments of the SM chain may melt at different temperatures,^40^ which is an interesting observation that could also be relevant in this system in terms of both differential segmental melting and differential melting of SM and DPPC. However, our experimental methods do not allow us to distinguish individual methylene groups, and this could be an informative future project.

**Figure 3:**
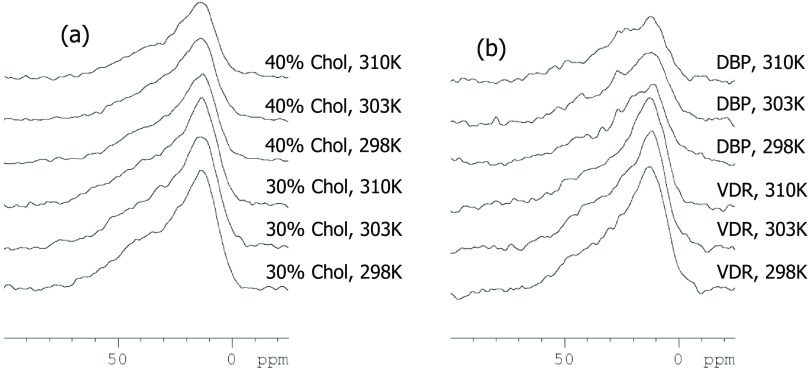
^31^P static NMR spectra of samples containing equimolar quantities of DPPC and bSM in 66 wt % water. (a) Effect of cholesterol (Chol) content and temperature. (b) Effect of adding protein (vitamin D receptor, VDR, or vitamin D binding protein, DBP) to samples with 30 mol % Chol at different temperatures.

^31^P NMR spectra confirm the presence of a lamellar phase based on the characteristic appearance of the CSA, as shown in Figure 3.

The anisotropic chemical shift tensor, Δσ, relates to the width of the asymmetric peak and reports on the phosphate group environment.^41^ The values of Δσ range from 40–48 ppm in all but one sample (30% Chol in the presence of DBP), for which a poor signal-to-noise ratio led to an inferior fit. These values are consistent with values reported previously for DPPC/Chol^42^ and for SM/Chol^43^ systems over comparable ranges of temperature and cholesterol content. The extracted values of η, which is the asymmetry parameter of the chemical shift tensor, are in the range 0.23–0.30. Given the single-nuclei fitting performed and the results of X-ray diffraction on the same samples, it is likely that the nonzero values of η are linked to overlapping CSAs from two lamellar phases with similar values of Δσ and/or the presence of an *L*_β_ phase.^44^ There is no substantial or consistent variation in Δσ or η on the addition of either DBP or VDR, although the spectra of the DBP sample have a somewhat poorer signal-to-noise ratio than the other samples (likely a sample-loading effect).

**Figure 4:**
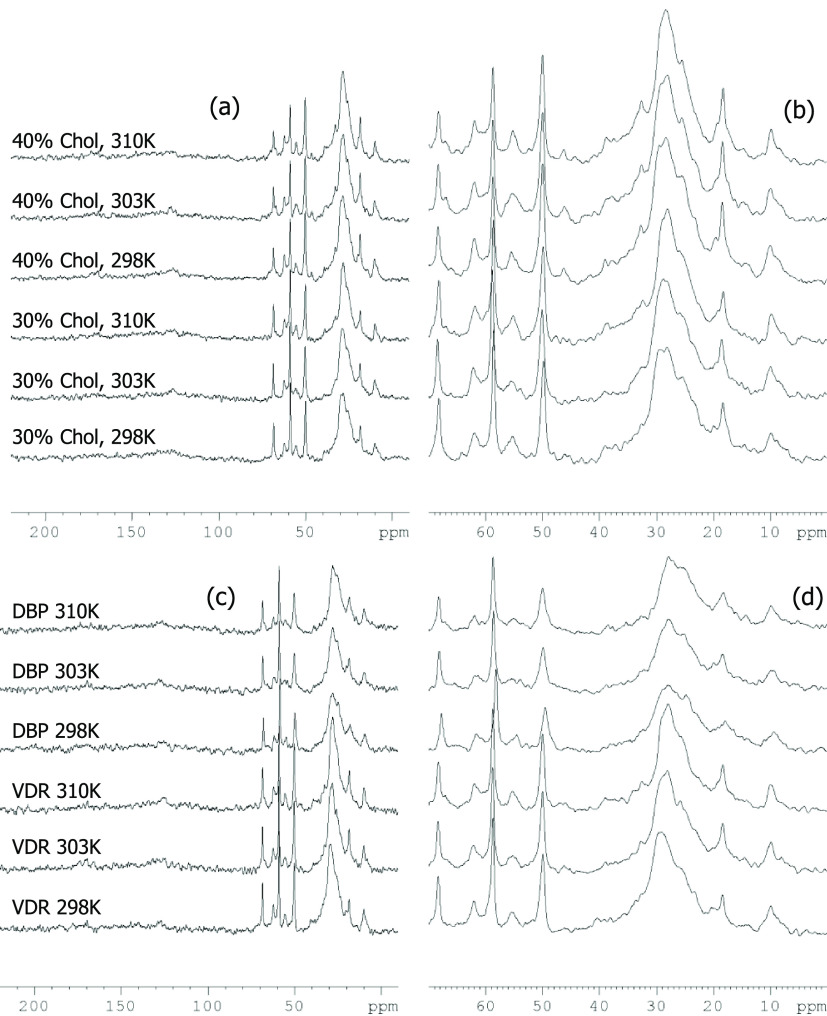
^13^C MAS NMR spectra of samples containing equimolar quantities of DPPC and bSM in 66 wt % water. (a) Effect of cholesterol (Chol) content and temperature, with expanded headgroup/methylene spectral region in panel (b) to show detail. (c) Effect of adding protein (vitamin D receptor, VDR, or vitamin D binding protein, DBP) to samples with 30 mol % Chol at different temperatures, with similarly expanded spectral region in panel (d) to show detail.

^13^C NMR MAS spectra are shown in Figure 4. Peaks arising from the choline headgroup and interfacial carbon atoms are visible in the region of 50–73 ppm, with the sharpest peaks arising from the choline *C*_γ_ (*N*-methyl) at 54.3 ppm, the choline *C*_α_ at ∼59.5 ppm, and the choline C_β_ at ∼66.3 ppm (see^45^ for choline carbon assignment).

The broad and complex peaks centered at ∼32 ppm result from the acyl chain methylene carbon atoms, probably with some contribution from Chol, and the sharper peak at 22 ppm can be assigned to acyl methylene carbon atoms close to the terminal methyl with a likely contribution from the C26 and C27 Chol methyl groups (see^30^ for Chol carbon numbering). The bulk acyl chain peaks centered around 32 ppm are similar to those reported for gel phase DPPC or eSM.^46^

The peak at 14 ppm (internally calibrated) can be ascribed to the terminal methyl groups of the lipid acyl chains. This methyl peak tends to be asymmetric (individual methyl groups from different lipid acyl chains are not resolved) and could also include a small contribution from the Chol C18 methyl carbon expected at ∼12 ppm. Peak assignments are made with reference to reported chemical shifts for Chol,^30^ SM with Chol,^47^ POPC with Chol,^48^ and other saturated diacylglycerol-phosphatidylcholines.^45^

There are some notable absences in the reported peaks. In particular, the phospholipid acyl/amide carbonyl resonances expected between 170 and 180 ppm are not well-resolved in any spectrum, and the alkene resonances expected for sphingosine, cholesterol, and any unsaturated component of bSM in the region 120–140ppm are not visible, although there is some distortion of the baseline in the 120–135 ppm range. Combined with the broadness and lack of systematic, temperature-dependent change in chemical shift of the bulk methylene peak,^38,49^ the main conclusion from these data is that the lipid interfacial (e.g., carbonyl) and acyl chain carbon atoms in these conditions experience motional restriction that is more similar to that associated with an L_β_ gel phase than with a more fluid *L*_α_ or *L*_o_ phase (e.g., see^47^ for a comparison of SM spectra above and below *T*_m_). Combined with the XRD data, we see no evidence of a clear gel–fluid *T*_m_ melting transition over the temperatures studied (up to 41 °C). This suggests that incorporation of Chol in the quantities studied has suppressed the melting transitions normally observed for single-lipid systems containing SM or DPPC at 39 or 42 °C.^24^ As with the ^31^P NMR data, there is no substantial or consistent difference between ^13^C NMR spectra of samples containing protein and those without.

The X-ray scattering data were further analyzed to extract approximations for the electron density profiles in the conditions studied. The electron density profiles were approximated with eq (1)
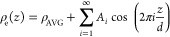
1where *ρ*_AVG_ is the average and, to a good approximation, constant electron density *d* is a repeat spacing, and *z* is the direction perpendicular to the model lipid membrane surface.

**Figure 5:**
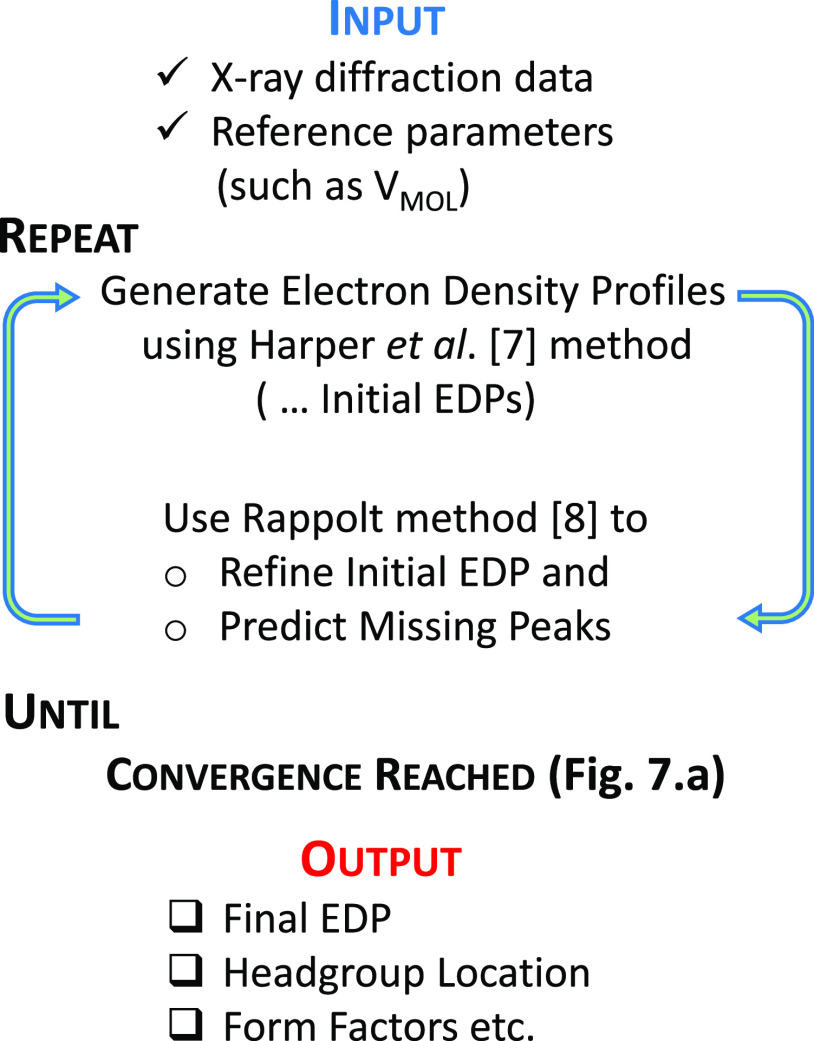
Pseudocode describing the self-consistent X-ray diffraction analysis process, where initial data and known molecular parameters^24^ are fed into electron density profile calculation, the output of which is then fed to form factor analysis. The process is iterated until a convergence in the position of headgroups is recorded.

**Figure 6:**
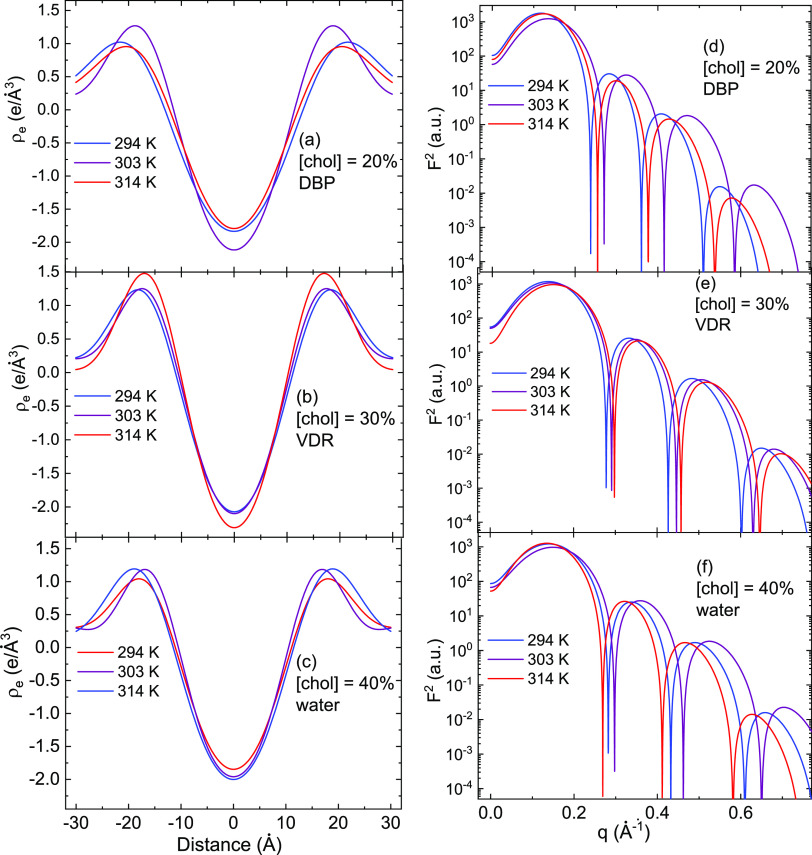
(a–c) Electron density profiles obtained based on initial raw data input and Harper et al.’s^7^ approach for DBP, VDR, and water at cholesterol concentrations of 20, 30, and 40%, respectively. Each panel contains density profiles at three temperature values. (d–f) Square of the form factor obtained following the approach by Rappolt^8^ for the same samples and experimental conditions.

Due to the data quality (number of reflections, peak broadness, and inhomogeneity), an iterative process was employed (see Figure 5 for pseudocode), and an example of the output of the analysis is shown in Figure 6. To generate the electron density profiles shown, one additional form factor was estimated beyond the measured number of reflections (so a total of 3 or 4 reflections were used for each profile). Form factors were calculated relying on eq (2)
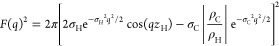
2where σ_H_ is the Gaussian width of the charge distribution at the hydrophilic heads, the peaks of which are located at positions +/– *z*_H_, *σ*_C_ is “negative” Gaussian width peaking at *z* = 0, the position representing the hydrophobic core of the model lipid bilayer, and *ρ*_C/_*ρ*_H_ is the electron density ratio.

With this model setup and constraints, the estimated headgroup positions seemed the most appropriate output, although these should be interpreted cautiously as an indication of the trends and the ranges of values rather than as exact values. Headgroup position (*z*_H_) was calculated from the model output as per reference (8).

Given the structure and the role these samples have, it is of interest to check the possible changes of the headgroup position as a function of the externally controlled parameters, such as temperature and cholesterol concentration. For convenience, we have organized the analysis so that the successive iterations between electron density profiles as well as form factors stop when the relative change in the position of the headgroup for a given sample and the set of experimental conditions does not exceed 1 part in 10^4^. We note that the results are the same even with more stringent requirements.

For the sake of clarity, we plotted only the last iteration for each case of experimental parameters in Figure 6 to show similarity with the previous result by Rappolt.^8^ Additional data are available that demonstrate the convergence.

We focused the analysis of the model output on the variations in the headgroup locations. We observe that for DBP at 294 K, the headgroup location changes by 17% over the course of cholesterol concentration change from 20 to 40%. For VDR, at the same temperature, the change is about 8%. There is less variation between samples at 40% Chol than at 30 or 20% Chol, supporting the possibility that the addition of Chol reduces heterogeneity in this system, as has been observed in other systems.

**Figure 7:**
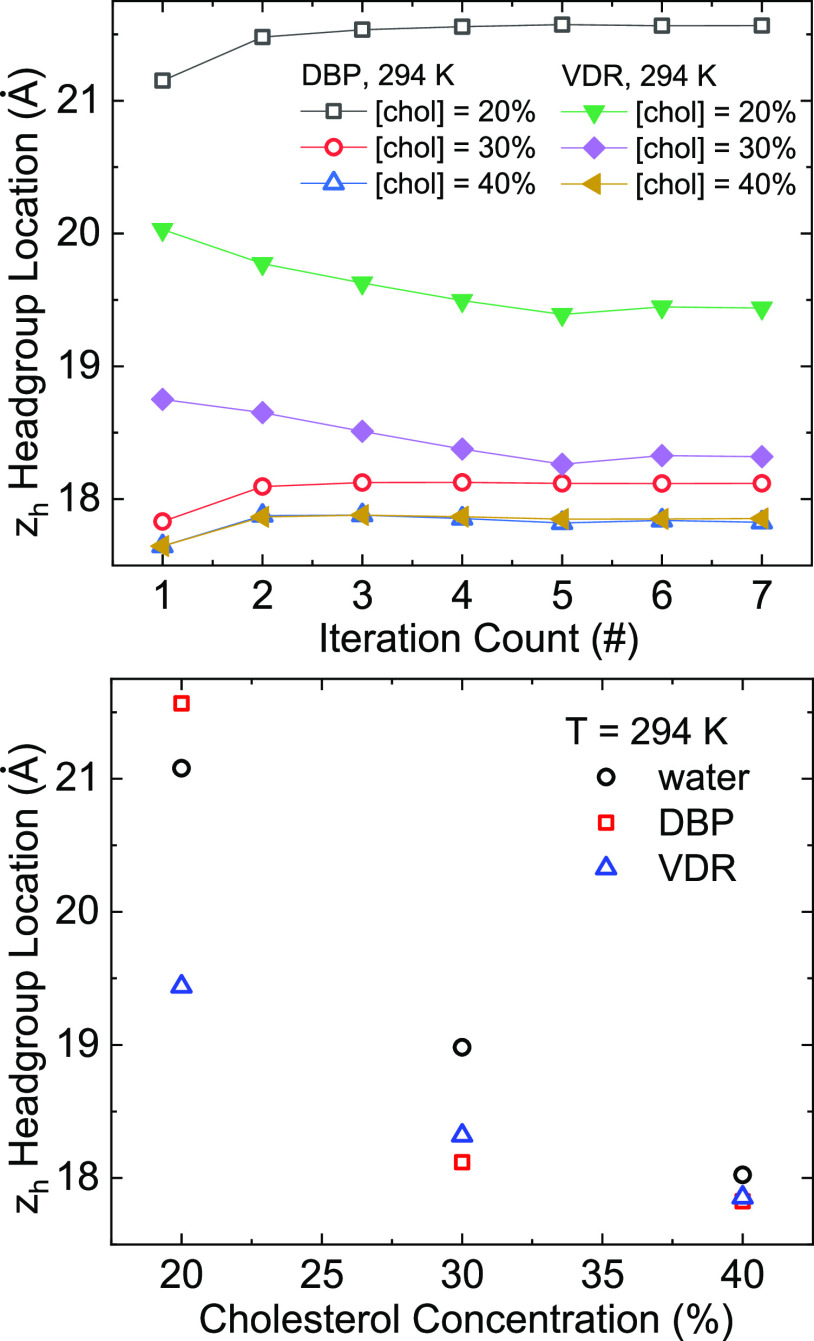
(Top) Variation of the headgroup locations across VDR and DBP samples for varied temperature and cholesterol concentrations. The uncertainty in all points is below 0.5 Å. In most cases, there are no significant variations after the 5th iteration of the self-consistent approach discussed above, but for the sake of thoroughness, we have checked samples up to the 10th iteration. (Bottom) The changes in the headgroup location as a function of the cholesterol concentrations for three samples at room temperature.

As expected, the general trend in the headgroup position is for bilayer thickness to decrease as cholesterol content increases. It is notable that there is more difference between the output for samples in the three different conditions (water, DBP, or VDR) at 20% Chol than at 30 or 40% Chol. As with a qualitative inspection of the X-ray scattering peaks, it is apparent that the samples displayed more heterogeneity at lower Chol content (Figure 7).

## Conclusions

XRD and NMR were used to investigate the structure of the DPPC/bSM/Chol ternary mixture of phospholipids, with the controlled parameters such as (a) vitamin D-relevant proteins (DBP and VDR); (b) physiological temperatures between 294 and 314 K; and (c) Chol concentrations between 20 and 40% by mol. wt. In general—and with some exceptions—increasing temperature tended to reduce the appearance of distinct shoulders in the small-angle region of the X-ray pattern and to result in larger and broader wide-angle peaks relating to acyl chain packing. Increasing Chol content had a similar effect, particularly at 40 mol %. These results are reflected in the general decrease in headgroup position with increasing Chol content, reflecting increased chain disorder and a concomitant reduction in bilayer thickness. Lipid carbonyl and acyl chain carbon atoms experience motional restriction that is much more likely to be of the expected behavior of *L*_β_ phase than either of the fluid phases, *L*_O_ or *L*_α_. Despite some reasonable suggestions in the literature, we do not see evidence for gel-to-fluid transition, as *T*_m_ appears to be above the physiological temperature range we probed. As might be expected given the low concentrations used, there was no significant effect of the vitamin D-relevant proteins.

### Prospective Views and Future Work

It is not clear to us why there is no change in the ^13^C methylene peak position, while the XRD suggests a measurable change in bilayer thickness and chain packing. It is possible that additional work on gel phase only would help clarify this.

Given the relatively rich variations in the structural details, we plan to analyze the same samples with X-ray beams significantly better focused (spot size of the order of 100 nm, as opposed to the 2–4 μm range used in the present study). This would likely help us resolve the size and dynamics of the likely domain structure.

Studies in a broader range of temperatures and possibly those done by differential scanning thermometry in addition to XRD and NMR would help elucidate details of the ternary phase diagram.

## Data Availability

Data from this manuscript are available upon reasonable request.

## Abbreviations Used

**CSA**: chemical shift anisotropy
**DBP**: Vitamin D binding protein
**DOPC**: 1,2-dioleoyl-*sn*-glycero-3-phosphocholine
**DPPC**: 1,2-dipalmitoyl-*sn*-glycero-3-phosphocholine
**DSC**: differential scanning calorimetry
***L***_**α**_: lamellar fluid disordered phase
***L***_**β**_: lamellar gel phase
***L***_**o**_: lamellar liquid-ordered phase
**LUV**: large unilamellar vesicle
**MAS**: magic angle spinning
**MLV**: multilamellar vesicle
**NMR**: nuclear magnetic resonance
**SM**: sphingomyelin
***T***_**m**_: phospholipid gel–fluid melting transition temperature
**VDR**: Vitamin D receptor
**XRD**: X-ray diffraction
